# Anti-inflammatory effect of antidiabetic thiazolidinediones prevents bone resorption rather than cartilage changes in experimental polyarthritis

**DOI:** 10.1186/ar2354

**Published:** 2008-01-16

**Authors:** Meriem Koufany, David Moulin, Arnaud Bianchi, Mikhaela Muresan, Sylvie Sebillaud, Patrick Netter, Georges Weryha, Jean-Yves Jouzeau

**Affiliations:** 1 Laboratoire de Physiopathologie et Pharmacologie Articulaires (LPPA), UMR 7561 CNRS-Nancy Université, avenue de la forêt de Haye, BP 184, 54505 Vandoeuvre-lès-Nancy, France; 2 Centre Hospitalier Régional et Universitaire, Service d'Endocrinologie/Médecine E, rue du Morvan, 54511 Vandoeuvre-lès-Nancy, France

## Abstract

**Background:**

Rosiglitazone and pioglitazone are high-affinity peroxisome proliferator-activated receptor (PPAR)-γ agonists with potent anti-diabetic properties and potential anti-inflammatory effects. We compared the ability of a range of oral doses of these thiazolidinediones, including those sufficient to restore insulin sensitization, to inhibit the pathogenesis of adjuvant-induced arthritis (AIA).

**Methods:**

AIA was induced in Lewis rats by a subcutaneous injection of 1 mg of complete Freund's adjuvant. Rats were treated orally for 21 days with pioglitazone 3, 10 or 30 mg/kg/day, rosiglitazone 3 or 10 mg/kg/day, or with vehicle only. The time course of AIA was evaluated by biotelemetry to monitor body temperature and locomotor activity, by clinical score and plethysmographic measurement of hindpaw oedema. At necropsy, RT-PCR analysis was performed on synovium, liver and subcutaneous fat. Changes in cartilage were evaluated by histological examination of ankle joints, radiolabelled sulphate incorporation (proteoglycan synthesis), glycosaminoglycan content (proteoglycan turnover) and aggrecan expression in patellar cartilage. Whole-body bone mineral content was measured by dual-energy X-ray absorptiometry.

**Results:**

The highest doses of rosiglitazone (10 mg/kg/day) or pioglitazone (30 mg/kg/day) were required to reduce fever peaks associated with acute or chronic inflammation, respectively, and to decrease arthritis severity. At these doses, thiazolidinediones reduced synovitis and synovial expression of TNF-α, IL-1β and basic fibroblast growth factor without affecting neovascularization or the expression of vascular endothelial growth factor. Thiazolidinediones failed to prevent cartilage lesions and arthritis-induced inhibition of proteoglycan synthesis, aggrecan mRNA level or glycosaminoglycan content in patellar cartilage, but reduced bone erosions and inflammatory bone loss. A trend towards lower urinary levels of deoxipyridinolin was also noted in arthritic rats treated with thiazolidinediones. Rosiglitazone 10 mg/kg/day or pioglitazone 30 mg/kg/day increased the expression of PPAR-γ and adiponectin in adipose tissue, confirming that they were activating PPAR-γ in inflammatory conditions, although an increase in fat mass percentage was observed for the most anti-arthritic dose.

**Conclusion:**

These data emphasize that higher dosages of thiazolidinediones are required for the treatment of arthritis than for restoring insulin sensitivity but that thiazolidinediones prevent inflammatory bone loss despite exposing animals to increased fatness possibly resulting from excessive activation of PPAR-γ.

## Introduction

Adjuvant-induced arthritis (AIA) in the rat is an experimental model reproducing some immunological aspects of rheumatoid arthritis (RA) such as genetic linkage and T-cell dependence [1], as well as several pathological features including chronic inflammation, involvement of peripheral joints, polysynovitis and secondary destruction of cartilage and bone [2]. Its relevance to the pathogenesis of RA is further supported by the demonstration that pro-inflammatory cytokines are highly expressed in the developing arthritic process [3] and that clinically relevant anti-cytokine therapy decreased arthritis severity when used alone [4,5] or in combination [6,7]. In addition, the AIA model reproduces most of the bone changes found in RA [1], including inflammatory bone loss, which has been linked to an increased risk of fracture [8]. Finally, the administration of osteoprotegerin, a decoy receptor, prevented cortical and trabecular bone loss in arthritic rats [9], suggesting that induction of osteoclast differentiation by receptor activator of nuclear factor κB ligand (RANKL) in inflammatory synovium could have a role [10]. This model is therefore suitable for the study of the anti-arthritic and bone protective effects of drugs thought to regulate cytokine expression at the gene level, such as peroxisome proliferator-activated receptor (PPAR)-γ agonists.

PPARs are ligand-inducible nuclear *trans*-acting factors belonging to the steroid receptors family [11]. Among the three characterized isotypes, PPAR-α is expressed essentially in tissues contributing actively to the catabolism of fatty acids (mainly in liver, and less markedly in brown fat, kidney, heart and skeletal muscle), where it regulates the expression of genes involved in fatty acid uptake and ω-oxidation or β-oxidation [12]. PPAR-α is also expressed in endothelial and vascular smooth muscle cells, as well as in macrophages and foam cells, where it contributes to the control of inflammation [13,14]. PPAR-β/δ is expressed ubiquitously and takes part in the reverse transport of cholesterol and the oxidation of fatty acids [15]. It has profound anti-obesity and anti-diabetic actions in animal models [16] and has also been linked to wound healing [17]. PPAR-γ is highly expressed in adipose tissue, where it has a pivotal role in adipocyte differentiation and lipid storage [12]. Its activation has been linked to insulin-sensitizing properties that have entered the clinics [14,18] and to the suppression of the release of cytokines, resulting in anti-inflammatory effects [19].

The anti-arthritic potency of PPAR agonists has been demonstrated only rarely in patients with RA [20]. In contrast, several studies have demonstrated the ability of PPAR agonists to decrease the severity of experimental polyarthritis [21] with a major effect on the expression of inflammatory genes [22-24] or on oxidative stress [22,24]. However, some data were obtained with 15-deoxy-Δ^12,14^-prostaglandin J_2 _[24,25], which is known to have anti-inflammatory properties independently of PPAR-γ activation [26]. Moreover, synthetic agonists were sometimes administered in a non-classical way such as the oral use of 10% dimethylsulphoxide as a vehicle [24] or repeated intraperitoneal administration [25], which could possibly interfere with the arthritic process [27]. Finally, daily doses of thiazolidinediones (TZDs) as high as 100 mg/kg/day were reported to be effective in experimental arthritis [21,25] although these doses are far above those required to restore insulin sensitivity. As glitazones are used primarily as antidiabetic agents, we decided to study the effects of rosiglitazone and pioglitazone on the arthritic process, cartilage changes and secondary bone loss when given orally at doses including those shown previously to be effective as insulin sensitizers [28-31].

In the present study we show that rosiglitazone 10 mg/kg/day or pioglitazone 30 mg/kg/day were required to decrease inflammation-induced fever and arthritis severity. At these anti-inflammatory doses, TZDs decreased synovitis and the expression of several cytokines and growth factors (TNF-α, IL-1 and basic fibroblast growth factor (bFGF)) without affecting neovascularization. However, none of the TZDs decreased proteoglycan changes in arthritic cartilage while preventing bone erosions and inflammatory bone loss. Both molecules induced PPAR-γ-dependent responses in adipose tissue but the maximal anti-arthritic effect was accompanied by increased fatness in animals. These data demonstrate that the anti-inflammatory potency of TZDs is of poor relevance to their insulin-sensitizing properties and suggest that a strong activation of PPAR-γ may expose arthritic patients to drawbacks secondary to excessive adipocyte differentiation.

## Materials and methods

### Animals

Ninety-three inbred male Lewis rats (Charles River, L'Arbresle, France) weighing 150 to 175 g were acclimated for 1 week in the laboratory before use. Animals were housed in groups of three or four in solid-bottomed plastic cages with free access to tap water and standard rodent pelleted chow (Scientific Animal Food & Engineering A04, Villemoisson-sur-orge, France) *ad libitum*. Room temperature was set at 23 ± 1°C and animals were subjected to a 12-hour light cycle (with light on from 06:00 to 18:00). All experiments were performed in accordance with national animal care guidelines and were pre-approved by a local ethics committee. Arthritis induction, implantation of biotelemetry sensors, blood sampling and necropsy were therefore performed under general anaesthesia, using volatile anaesthetics (AErrane™; Baxter SA, Maurepas, France).

### Induction of arthritis and treatment regimen

Arthritis was induced on day 0 at the basis of the tail by a single subcutaneous injection of 0.1 ml of a suspension containing 10 mg/ml heat-inactivated *Mycobacterium tuberculosis *H37Ra (Difco Laboratory, Detroit, MI, USA) emulsified in a sterile mixture of paraffin oil, saline and Tween 80. Naive animals served as controls (normal controls).

Animals were randomly assigned to one of the following treatment groups: arthritic untreated controls (AIA controls), arthritic rats treated with rosiglitazone (3 or 10 mg/kg/day) and arthritic rats treated with pioglitazone (3, 10 or 30 mg/kg/day). Treatment was given from the day of sensitization until necropsy (day 21). Thiazolidinediones were administered once a day by gastric gavage as a suspension in 0.5% carboxymethylcellulose at a dose of 1 ml per 100 g body weight. Treatment was prepared daily from marketed pills of Avandia™ (Glaxo-Smith-Kline, Marly-le-Roy, France) and Actos™ (Takeda, Puteaux, France). Naive rats (normal controls) and AIA controls received carboxymethylcellulose only.

### Assessment of arthritis

#### Body weight

Total body weight was recorded every other day from day 6 to day 21. At the indicated times, the increase in body weight was calculated relative to that at day 0 allowing monitoring of the decrease in body weight gain associated with arthritis.

#### Arthritic score

Animals were scored regularly until day 21 by two investigators who were blind to the treatment. Each paw was graded according to the severity and extent of erythema and swelling of periarticular soft tissues, and the enlargement and distortion of the joints [32]. Clinical score ranged from 0 (no sign) to 4 (severe lesions), yielding a maximum score of 16 per animal.

#### Hindpaw oedema

Swelling of both hindpaws was measured regularly until day 21 by plethysmography. In brief, hindpaw volume was measured up to the skin–coat junction of the rear footpad through the displacement of an equivalent volume of water in a plethysmometer 7150 (Apelex, Massy, France). At the indicated times, paw volume was compared with the basal level (day 0) and oedema was expressed as volume change (ml).

### Evaluation of arthritis time course by biotelemetry

Body temperature and locomotive activity were monitored hourly between 18:00 and 06:00 (dark cycle of nocturnal intense activity) and recorded from day -1 (nocturnal data control) to day 17 with battery-operated biotelemetry devices (Mini-Metter, model VMHF; Paris, France) implanted into the peritoneal cavity [33]. In brief, the implanted sensor generates radio frequency waves that are modulated by the waves radiating from the animal (depending on body temperature) and are detected by a receiver placed beneath the animal's cage. Mobility is measured as pulses corresponding to signal strengths generated by changes in the orientation of the implanted transmitter relative to the T antenna of the receiver. Signals are relayed by a consolidation matrix into a peripheral processor connected to a computer. Fever was expressed as the daily difference in the mean nocturnal temperature relative to the mean nocturnal temperature recorded before sensitization (day -1). The activity index was expressed as the daily percentage of the mean nocturnal activity relative to the control mean nocturnal activity (day -1), with a negative value representing a loss of spontaneous mobility. For each treatment group, data were further expressed as the area under the curve over the time course of the primary phase (days 0 to 3) and the secondary phase (days 4 to 17) of arthritis.

### Assessment of proteoglycan metabolism in patellar cartilage

#### Proteoglycan synthesis

Proteoglycan synthesis was studied by an *ex vivo *incorporation of Na_2_^35^SO_4 _into patellar cartilage. At necropsy, patellas were collected aseptically, dissected from periarticular tissues, then pulsed for 3 hours at 37°C in a 5% CO_2 _atmosphere with 0.6 μCi/ml Na_2_^35^SO_4 _(Amersham, Les Ulis, France) in RPMI-Hepes 1640 medium supplemented with 2 mM L-glutamine,100 IU/ml penicillin and 100 μg/ml streptomycin (Life Technologies, Cergy-Pontoise, France). After five washings in saline, patellas were fixed overnight in 0.5% cetylpyridinium chloride (Sigma, Saint Quentin-Fallavier, France) in 10% (v/v) phosphate-buffered formalin, then decalcified in 5% (v/v) formic acid for 6 hours at room temperature. Biopsy punches, 2 mm in diameter, were taken from the central part of the patellas before dissolution overnight in Soluene 350 (Packard, Rungis, France). ^35^S-proteoglycan content was measured by liquid scintillation counting (Hionic Fluor; Packard, Rungis, France) and data are expressed as the percentage variation from healthy controls, with a negative value representing a decrease in proteoglycan synthesis.

#### Glycosaminoglycans content

Sulphated glycosaminoglycan content was evaluated in patellar cartilage with the 1,9-dimethylmethylene blue (Sigma-Aldrich, Saint Quentin-Fallavier, France) colorimetric assay [34]. In brief, patellas were decalcified overnight in 5% (v/v) formic acid at room temperature before separation of cartilage layer from underlying bone. Cartilage was dried for 1 day at room temperature, weighed on a high-precision balance (± 0.01 mg), then hydrolysed for 4 hours at 60°C with 60 μg (0.6 IU) of papain (Sigma, Saint Quentin-Fallavier, France) in enzymatic buffer (2 mM dithiothreitol, 1 mM EDTA, 20 mM Na_2_HPO_4_). Hydrolysis was stopped by the addition of iodoacetate sodium salt (10 mM final concentration) before neutralization with Tris-HCl buffer pH 8.0. The assay was performed by monitoring the metachromatic reaction of sulphated glycosaminoglycans with 1,9-dimethylmethylene blue at 525 nm, with chondroitin 6-sulphate (Institut Jacques Boy, Reims, France) as a standard. The calibration curve ranged from 0 to 100 μg/ml chondroitin 6-sulphate, and data are expressed as μg of glycosaminoglycan per mg of cartilage.

### Histological analysis

Ankle and knee joints were collected at necropsy, fixed immediately for 24 hours in 4% paraformaldehyde, then decalcified in rapid bone decalcifier (RDO; Apex Engineering, Plainfield, IL, USA) for 6 hours at room temperature, and further fixed in 4% paraformaldehyde before embedding in paraffin. Sections (5 μm thick) were rehydrated in a graded ethanol series and stained with haematoxylin/eosin/safran and toluidine blue (ankle joint) or May Grunwald Giemsa (knee joint).

The histological characteristics of ankle articular cartilage, bone and periarticular soft tissue were scored by a blinded observer. Cartilage degradation was graded from 0 to 3, where 0 = fully stained cartilage, 1 = destained cartilage, 2 = destained cartilage with synovial cells invasion, and 3 = complete loss of cartilage [35]. The following morphological criteria were used for bone erosion: 0 = normal, 1 = mild loss of cortical bone at few sites, 2 = moderate loss of cortical and trabecular bone, and 3 = marked loss of bone at many sites [36].

Synovium from ankle joint was graded using a scoring technique adapted from Rooney and colleagues [37]. In brief, samples were evaluated on a scale of 0 to 4 (from 0 = normal to 4 = major changes) for hyperplasia of synovial fibroblasts (depth of lining layer), fibrosis (percentage replacement of loose connective tissue), focal aggregates of lymphocytes (percentage aggregate around the lining layer), angiogenesis (number of proliferating blood vessels), perivascular infiltrates of lymphocytes (percentage of vessels surrounded by lymphocytes) and tissue infiltration by lymphocytes (size of aggregates, percentage infiltrating cells). For each group, four or five sections were taken and graded at different fields to provide a representative sample of the whole joint. Mean scores were determined from the different sections of the individual animals, allowing the calculation of composite scores for the different experimental groups.

### Analysis of gene expression

#### RNA isolation

Tibial plateaux, articular fat pad, liver, and peritoneal adipose tissue were collected aseptically at necropsy and processed for RNA isolation. Tibial plateaux were decalcified for 12 hours with 165 mM EDTA pH 7.4 in RNA Later™ (Ambion, Huntingdon, UK) before separation of the cartilage layer from the underlying bone. Total RNA was extracted from decalcified cartilage and frozen tissues by grinding in Trizol™ solution (Sigma, St Quentin-Fallavier, France). The integrity of the RNA pool was verified by electrophoresis in agarose gel containing 0.5 μg/ml ethidium bromide.

#### Gene amplification by PCR

Total RNA (2 μg) was reverse transcribed for 1 hour at 37°C with 200 U of Moloney murine leukaemia virus reverse transcriptase (Gibco BRL, Cergy-Pontoise, France) using random hexamer primers (100 pmol) (MWG biotech SA, Courtaboeuf, France).

In fat pad, PCR amplification was performed on an aliquot of RT products diluted 10× by *Taq *polymerase (2.5 U; Gibco BRL, Cergy Pontoise, France) and specific primers (MWG biotech SA, Courtaboeuf, France) (Table 1). The conditions for amplification were: denaturation at 94°C for 45 s, hybridization of primers at a defined temperature for 45 s, and elongation at 72°C for 45 s. The numbers of amplification cycles were chosen in the exponential phase of PCR. PCR products were analysed by electrophoresis in 2% agarose gel containing 0.5 μg/ml ethidium bromide, and quantification was performed with Geldoc 2000™ software (Bio-Rad, Marnes-la-Coquette, France). The housekeeping gene encoding the ribosomal protein L27 was used as an internal control, and results were expressed as the normalized ratio of mRNA level of each gene of interest over the gene encoding L27.

**Table 1:** Primers used for semi-quantitative PCR and product length

Gene encoding	Primer sequence	Size (base pairs)	Cycles	*T*_m _(°C)
L27	Sense: 5'-TCCTGGCTGGACGCTACTC-3'	225	27	62
	Antisense: 5'-CCACAGAGTACCTTGTGGGC-3'			
MCP-1	Sense: 5'-ATGCAGTTAATGCCCCACTC-3'	167	29	57
	Antisense: 5'-TTCCTTATTGGGGTCAGCAC-3'			
bFGF	Sense: 5'-GAACCGGTACCTGGCTATGA-3'	182	31	61
	Antisense: 5'-CCGTTTTGGATCCGAGTTTA-3'			
VEGF	Sense: 5'-CAATGATGAAGCCCTGGAGT-3'	211	32	64
	Antisense: 5'-TTTCTTGCGCTTTCGTTTTT-3'			
TNF-α	Sense: 5'-AGATGTGGAACTGGCAGAGG-3'	178	31	58
	Antisense: 5'-CCCATTTGGGAACTTCTCCT-3'			
IL-1β	Sense: 5'-TGAAAGCTCTCCACCTCAATGG-3'	366	28	61
	Antisense: 5'-TCCATGGTGAAGTCAACTATGTCC-3'			

MCP-1, monocyte chemotactic protein-1; bFGF, basic fibroblast growth factor; VEGF, vascular endothelial growth factor; *T*_m_, melting temperature.

In other tissues, real-time polymerase chain reaction analysis was performed with LightCycler™ technology (Roche Diagnostics, Basel, Switzerland) and SYBRgreen master mix system™ (Qiagen, Courtaboeuf, France). After amplification with specific primers (Table 2), a melting curve was performed to determine the melting temperature of each PCR product. Product sizes were controlled on a 2% agarose gel stained with 0.5 μg/ml ethidium bromide. Each run included standard dilutions and positive and negative reaction controls. mRNA levels of each gene of interest and of the ribosomal protein RP29, chosen as a housekeeping gene, were determined for each sample. Results were expressed as the normalized ratio of the mRNA level of each gene of interest over the gene encoding RP29.

**Table 2:** Primers used for real-time PCR and product length

Gene encoding	Primer sequence	Size (base pairs)	*T*_m _(°C)
RP29	Sense: 5'-AAGATGGGTCACCAGCAGCTCTACTG-3'	67	59
	Antisense: 5'-AGACGCGGCAAGAGCGAGAA-3'		
Aggrecan	Sense: 5'-ACACCCCTACCCTTGCTTCT-3'	124	58
	Antisense: 5'-AAAGTGTCCAAGGCATCCAC-3'		
PPAR-α	Sense: 5'-GATGACCTGGAAAGTCCCTT-3'	59	56
	Antisense: 5'-CTTGAATGTTTCCCATCTCTT-3'		
PPAR-γ	Sense: 5'-ATGGGTGAAACTCTGGGAGAT-3'	92	56
	Antisense: 5'-GGTAATTTCTTGTGAAGTGCT-3'		
Adiponectin	Sense: 5'-AATCCTGCCCAGTCATGAAG-3'	433	58
	Antisense: 5'-TCTCCAGGAGTGCCATCTCT-3'		
ACO	Sense: 5'-CCAATCACGCAATAGTTCTGG-3'	362	57
	Antisense: 5'-CGCTGTATCGTATGGCGAT-3'		

PPAR, peroxisome proliferator-activated receptor; ACO, acyl-CoenzymeA oxidase; *T*_m_, melting temperature.

### Bone mineral density

Bone mineral density was determined *in vivo *by dual-energy X-ray absorptiometry (DEXA) with a model QDR-4500A densitometer (Hologic Inc., Waltham, MA, USA) and a small-animal module. Rats were anaesthetized as mentioned above, placed in a supine decubitus position with abduction of the four limbs, and scanned both on the day before arthritis induction (day -1) and on the day before necropsy (day 20). Each animal was scanned five times consecutively after repositioning, bone mineral density measurement being expressed as mean ± SD for a single time point. Bone mineral density (g/cm^2^) and bone mineral content (BMC, in grams) were determined on the whole body (total BMC), each measurement being performed by the same investigator, who was blind to the treatment. Data were expressed as changes in BMC and percentage of fat mass over the study duration, each animal being used as its own control. Internal variations of repeated measures of total rat bone mineral density have been determined to be between 1.5% and 2.0%.

### Biochemical markers of bone turnover

#### Plasma osteocalcin level

Heparinized plasma samples were collected on the day before sensitization (day -1) and at necropsy (day 21) by sampling veins of the tail and by cardiac puncture, respectively. Plasma osteocalcin concentration was measured with a sandwich enzyme-linked immunosorbent assay kit (Biomedical Technologies Inc., Stoughton, MA, USA). This assay is specific for rat osteocalcin, with a sensitivity of 0.5 ng/ml. Frozen heparinized samples were thawed once and diluted 1:10 to 1:20 with sample buffer in accordance with the manufacturer's recommendations. Data are expressed as changes in plasma osteocalcin concentration (ng/ml) over the study duration (day 21 minus day -1), each animal being used as its own control.

#### Deoxypyridinoline urinary level

Urinary deoxypyridinoline concentration was measured on the day before arthritis induction (day -1 to day 0) and the day before necropsy (day 20 to day 21), with a competitive enzyme immunoassay kit (Metra Biosystems, Palo Alto, CA, USA). This assay is specific for free deoxypyridinoline, with a sensitivity of 1.1 nmol/l; it shows acceptable cross-reactivity between animal species [38]. Spontaneous urine samples were collected over 24 hours without preservative by placing animals in metabolic cages. Frozen urine samples were thawed once and diluted 1:200 with assay buffer to measure against the standard curve. Urinary creatinine concentrations (mmol/l) were determined in parallel with a colorimetric assay kit (Metra Biosystems, Palo Alto, CA, USA) and served to correct deoxypyridinoline values for variation in urine concentration. Data are expressed as changes in deoxypyridinoline/creatinine concentration (nmol/mmol) over the study duration (day 21 minus day 0), each animal being used as its own control.

### Statistical analysis

Data are expressed as means ± SEM. Arthritis score and histological grading were analysed with the Mann–Whitney *U *test, using StatView™ version 5.0 software (SAS Institute Inc., Cary, NC, USA). All other data were compared by analysis of variance (ANOVA) followed by Fisher's protected least-squares difference (PLSD) post-hoc test. Differences were considered significant at *P *< 0.05 (*, *P *< 0.05 compared with normal controls;^#^, *P *< 0.05 compared with AIA controls).

## Results

### Dose–response study with glitazones

#### Effect of rosiglitazone and pioglitazone on arthritis incidence

As shown in Table 3, arthritis occurred in all animals sensitized with complete Freund's adjuvant. Treatment with a range of doses of rosiglitazone or pioglitazone did not reduce arthritis incidence in three separate experiments, suggesting that PPAR-γ agonists did not impair the immunological spreading of the disease.

**Table 3:** Effect of PPAR-γ agonists on incidence of adjuvant arthritis

Group	Experiment		
	
	1	2	3
AIA	5/5	6/6	7/7
AIA + ROSI 3	5/6	-	-
AIA + ROSI 10	5/5	7/7	8/8
AIA + PIO 3	6/6	-	-
AIA + PIO 10	6/6	-	-
AIA + PIO 30	6/6	6/7	8/8

PPAR, peroxisome proliferator-activated receptor; AIA, adjuvant-induced arthritis; ROSI, rosiglitazone 3 or 10 mg/kg/day; PIO, pioglitazone 3 or 10 or 30 mg/kg/day. Results are disease incidences at day 21.

#### Effect of rosiglitazone and pioglitazone on gain in body weight

**Figure 1:**
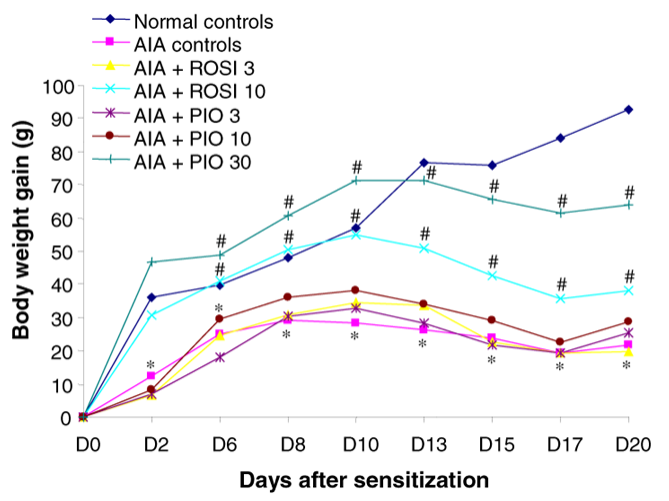
Modulation of body weight gain by rosiglitazone and pioglitazone in the course of adjuvant-induced arthritis. Male Lewis rats were sensitized subcutaneously on the basis of the tail with a single injection of 1 mg of *M. tuberculosis*. Animals were treated daily with 3 mg/kg (*n *= 6) or 10 mg/kg (*n *= 12) of rosiglitazone (ROSI) or 3 mg/kg (*n *= 6), 10 mg/kg (*n *= 6) or 30 mg/kg (*n *= 12) of pioglitazone (PIO) by oral administration. Arthritic (adjuvant-induced arthritis (AIA)) (*n *= 11) and normal controls (*n *= 10) were given 0.5% carboxymethylcellulose alone. Data are expressed as means ± SEM. *, *P *< 0.05 compared with normal controls; ^#^, *P *< 0.05 compared with AIA controls (ANOVA and Fisher's PLSD post-hoc test). D*n*, day *n*.

In our experimental conditions, the body weight of naive animals increased gradually, with a mean gain of about 4 to 5 g/day over the study duration (Figure 1). In all arthritic rats, body weight peaked at day 10, then decreased progressively as arthritis settled. The rate of change in body weight was similar in arthritic controls and in rats treated with 3 mg/kg/day of rosiglitazone, or 3 or 10 mg/kg/day of pioglitazone. The decrease in body weight gain was significantly lower from day 13 to day 20 in arthritic animals receiving 10 mg/kg/day of rosiglitazone or 30 mg/kg/day of pioglitazone. However, before the onset of arthritis, rats treated with 30 mg/kg/day of pioglitazone had a higher weight gain than normal controls. These data demonstrate that the highest doses of PPAR-γ agonists prevented arthritis-induced body weight loss, although being able to favour overweight independently of the arthritic process.

#### Effect of rosiglitazone and pioglitazone on the course of experimental arthritis

**Figure 2:**
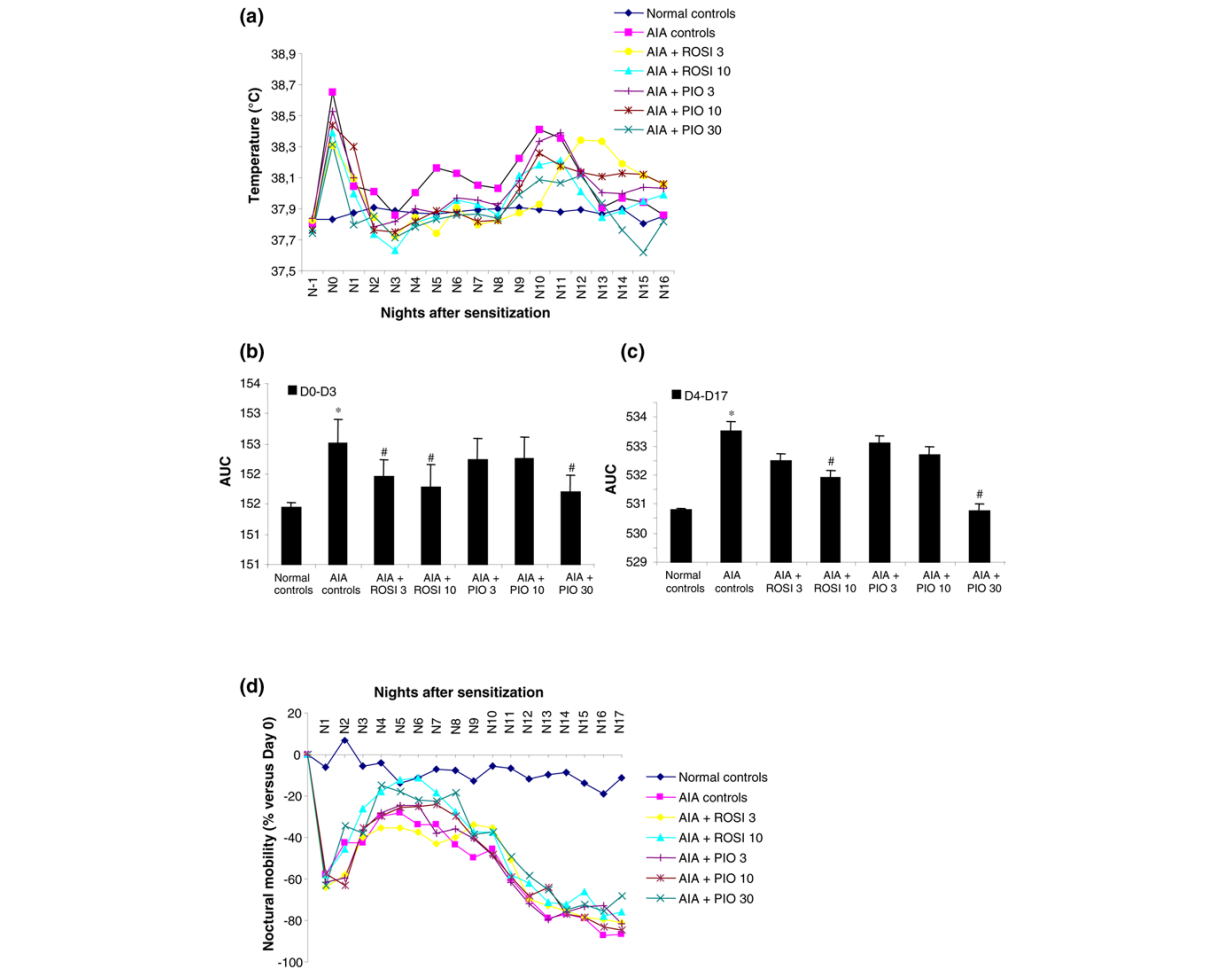
Modulation of body temperature and locomotive activity by rosiglitazone and pioglitazone treatment during adjuvant-induced arthritis. Animals were treated daily with 3 or 10 mg/kg of rosiglitazone (ROSI) or 3, 10 or 30 mg/kg of pioglitazone (PIO) by oral administration. Effect on **(a) **mean nocturnal body temperature, **(b) **primary inflammation, expressed as area under the time curve (AUC) of body temperature from day 0 to day 3 after sensitization, **(c) **secondary immunological inflammation, expressed as area under the time curve (AUC) of body temperature from day 4 to day 17 after sensitization, and **(d) **mean locomotive activity. Data are expressed as means ± SEM for at least five animals (ROSI 3, PIO 3 and 10) or 10 animals (normal controls, adjuvant-induced arthritis (AIA) controls, ROSI 10 and PIO 30). *, *P *< 0.05 compared with normal controls; ^#^, *P *< 0.05 compared with AIA controls (ANOVA and Fisher's PLSD post-hoc test). D*n*, day *n*; N*n*, night *n*.

The monitoring by biotelemetry showed that arthritic animals had a biphasic response in their body temperature. An early peak of fever appeared on day 1, secondary to the local acute inflammation induced by sensitization, followed by a return to the control level within 3 days (Figure 2a). A delayed peak of fever occurred from day 9, when the systemic phase of the arthritic response begun, but it was less intense than the primary peak (Figure 2a). Body temperature returned to normal levels within 5 days and remained stable until the end of the experiment. Arthritis-induced fever peaks were reduced variably by PPAR-γ agonists. Rosiglitazone had a moderate inhibitory effect on early fever at 3 mg/kg/day (Figure 2a,b), whereas it reduced both fever peaks at 10 mg/kg/day (Figure 2a,b,c). Pioglitazone was ineffective at 3 or 10 mg/kg/day (Figure 2a,b,c). However, it reduced early fever and, more importantly, delayed fever peak at 30 mg/kg/day (Figure 2a,b,c).

The monitoring of spontaneous locomotive activity showed that arthritic animals exhibited two successive losses of mobility (Figure 2d). The first period of hypomobility appeared from day 1 (-60%) after sensitization, with a partial recovery until day 4 (-30%). This time course was consistent with the early peak of fever and probably originated from the acute inflammation induced by sensitization. A secondary loss of mobility occurred from day 5 and worsened progressively until day 17 (Figure 2d). Contrary to the delayed peak of fever, secondary hypomobility was not transient and resulted in a major functional disability (-80% at day 17). Both rosiglitazone and pioglitazone were ineffective on both primary and secondary loss of mobility whatever the dosage used.

### Anti-arthritic potency of glitazones

#### Clinical parameters

**Figure 3:**
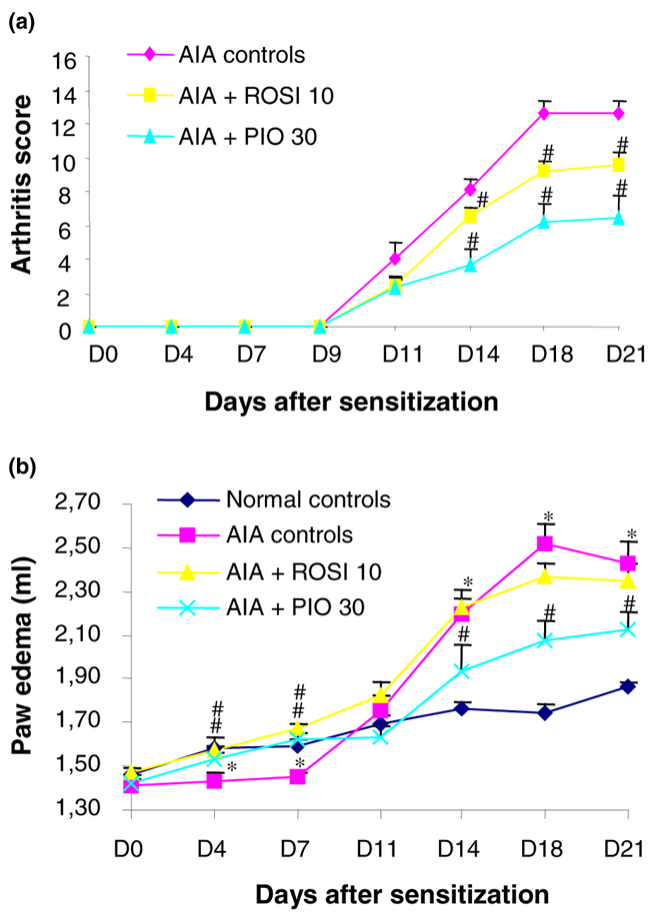
Modulation of disease severity by rosiglitazone and pioglitazone treatment during adjuvant-induced arthritis. Animals were treated daily with rosiglitazone (ROSI) 10 mg/kg (*n *= 8) or pioglitazone (PIO) 30 mg/kg (*n *= 8) by oral administration. Arthritic (adjuvant-induced arthritis (AIA)) (*n *= 7) and normal controls (*n *= 7) were given 0.5% carboxymethylcellulose alone. Arthritis score **(a) **and paw oedema **(b) **were assessed three times a week after onset of arthritis. For paw volume, each data point represents the mean of both hind paws. Data are expressed as means ± SEM. *, *P *< 0.05 compared with normal controls; ^#^, *P *< 0.05 compared with AIA controls (Mann–Whitney *U *test (arthritis score) or ANOVA and Fisher's PLSD post-hoc test (oedema)). D*n*, day *n*.

As shown in Figure 3, arthritis became obvious 11 days after sensitization and was maximal by day 18. Arthritis was severe: the mean arthritic score averaged 13 in untreated controls, highlighting the fact that animals had at least three arthritic paws (Figure 3a). Arthritis severity was reduced from day 14 by both PPAR-γ agonists, reaching an improvement of 23% for 10 mg/kg/day of rosiglitazone and 49% for 30 mg/kg/day of pioglitazone on day 21 (Figure 3a). Although paw volume increased progressively with the age of animals, bilateral hindpaw swelling was observed from day 14 in arthritic rats (Figure 3b). Contrary to the arthritic score, rosiglitazone was marginally effective at 10 mg/kg/day (-14% on day 21), whereas 30 mg/kg/day of pioglitazone reduced oedema by -54% on day 21 (Figure 3b).

#### Synovitis

**Figure 4:**
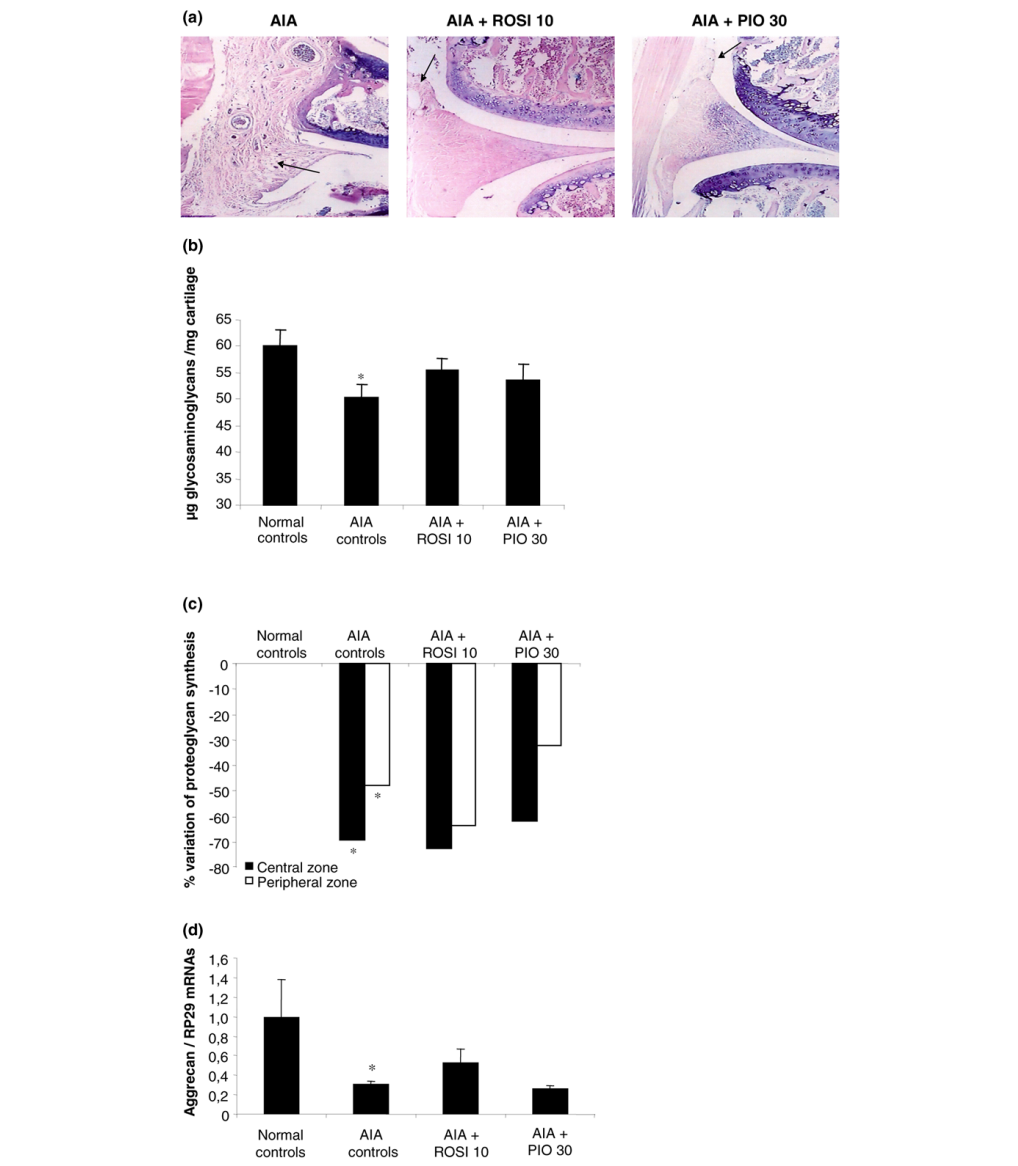
Effect of rosiglitazone and pioglitazone treatment on cartilage changes in arthritic knees. Animals were treated daily for 21 days with rosiglitazone (ROSI) 10 mg/kg (*n *= 8) or pioglitazone (PIO) 30 mg/kg (*n *= 8) by oral administration. Control animals with adjuvant-induced arthritis (AIA) (*n *= 7) and normal controls (*n *= 7) were given 0.5% carboxymethylcellulose alone. **(a) **A representative frontal section of the knee joint showing synovial membrane hyperplasia (MGG [May Grunwald Giemsa] staining, day 21 after sensitization). **(b, c) **Changes in proteoglycan metabolism in patellar cartilage: (b) sulphated glycosaminoglycan content by the 1,9-dimethylmethylene blue method expressed as μg of glycosaminoglycan per mg of cartilage. Data are expressed as means ± SEM; (c) radiolabelled sulphate incorporation expressed as mean percentage of normal controls. **(d) **Expression of aggrecan mRNA level normalized to RP29 in cartilage from tibial plateaux (RT-quantitative polymerase chain reaction). Data are expressed as means ± SEM of 4 animals per group. *, *P *< 0.05 compared with normal controls (ANOVA and Fisher's PLSD post-hoc test).

Histological examination of knee joints: overall histological examination of knee sections from arthritic controls showed a significant pannus invasion, along with infiltration by mononuclear cells and fibrosis, and a slight formation of new blood vessels (Figure 4a). Cellular infiltration was markedly decreased in rats treated with one or other glitazone, and no pannus formation was observed in these conditions (Figure 4a).

Expression of pro-inflammatory genes in fat pad: RT-PCR analysis showed overexpression of the pro-inflammatory cytokines TNF-α and IL-1β, of the angiogenic factor vascular endothelial growth factor (VEGF), of the growth factor bFGF and the chemokine monocyte chemotactic protein-1 (MCP-1) in fat pads of arthritic controls (Table 4). Expression of these mediators was not significantly affected in animals receiving 10 mg/kg/day of rosiglitazone, whereas mRNA levels of IL-1β, TNF-α and bFGF were decreased by 63%, 77% and 63%, respectively, in rats treated with 30 mg/kg/day of pioglitazone (Table 4). VEGF and MCP-1 mRNA levels were not significantly affected in these animals.

**Table 4:** Effect of PPAR-γ agonists on inflammatory genes levels in the knee synovium of arthritic rats

Groups	IL-1	TNF-α	VEGF	bFGF	MCP-1
Normal controls	0.23 ± 0.20	0.13 ± 0.07	1.39 ± 0.01	0.41 ± 0.18	0.26 ± 0.14
AIA controls	1.40 ± 0.29*	1.19 ± 0.19*	3.40 ± 1.05*	5.24 ± 0.35*	1.05 ± 0.18*
AIA + ROSI 10	0.98 ± 0.06	1.39 ± 0.16	3.79 ± 0.28	4.59 ± 0.77	1.51 ± 0.22
AIA + PIO 30	0.66 ± 0.16^#^	0.37 ± 0.11^#^	4.67 ± 0.21	2.18 ± 0.61^#^	1.51 ± 0.21

PPAR, peroxisome proliferator-activated receptor; AIA, adjuvant-induced arthritis; ROSI 10, rosiglitazone 10 mg/kg/day; PIO 30, pioglitazone 30 mg/kg/day; VEGF, vascular endothelial growth factor; bFGF, basic fibroblast growth factor; MCP-1, monocyte chemotactic protein-1. Data are expressed as means ± SEM of mRNAs of the gene of interest over L27 mRNA (*n *= 3 representative samples per group).

*, *P *< 0.05 compared with normal controls; ^#^, *P *< 0.05 compared with AIA controls (ANOVA and Fisher's PLSD test).

Histological grading of ankle joints: histological examination of ankle sections from arthritic controls showed a massive hyperplasia of synovial fibroblasts, with focal aggregates of lymphocytes and fibrosis (Table 5). A significant proliferation of blood vessels occurred in the inflamed synovial tissue, with a moderate perivascular and a marked diffuse infiltration by lymphocytes. Lesions were more severe than in corresponding knee joints, which is consistent with the distal spreading of the disease. Treatment with 10 mg/kg/day of rosiglitazone or 30 mg/kg/day of pioglitazone decreased synoviocyte hyperplasia, fibrosis, focal aggregates and diffuse infiltrates of lymphocytes, without modifying vessel-related events (angiogenesis and perivascular infiltration; Table 5).

**Table 5:** Effect of PPAR-γ agonists on the histological grading of ankle synovium in arthritic rats.

Parameter	Normal controls	AIA controls	AIA + ROSI 10	AIA + PIO 30
Synoviocyte hyperplasia	0 ± 0	3.93 ± 0.12*	2.00 ± 0.42^#^	0.80 ± 0.19^#^
Focal aggregates of lymphocytes	0.08 ± 0.14	3.50 ± 0.24*	1.60 ± 0.23^#^	1.20 ± 0.19^#^
Fibrosis	0.37 ± 0.26	3.73 ± 0.20*	2.60 ± 0.31^#^	1.00 ± 0.30^#^
Proliferating blood vessels (original magnification × 40)	0.37 ± 0.26	1.50 ± 0.24*	0.80 ± 0.35	1.07 ± 0.12
Perivascular infiltrates of lymphocytes	0.62 ± 0.26	1.60 ± 0.31*	0.80 ± 0.35	1.10 ± 0.25
Diffuse infiltrates of lymphocytes	0 ± 0	3.13 ± 0.16*	2.10 ± 0.25^#^	0.70 ± 0.22^#^

PPAR, peroxisome proliferator-activated receptor; ROSI 10, rosiglitazone 10 mg/kg/day; PIO 30, pioglitazone 30 mg/kg/day; AIA, adjuvant-induced arthritis. Data are expressed as means ± SEM (*n *= 5 representative animals per group).

*, *P *< 0.05 compared with normal controls; ^#^, *P *< 0.05 compared with AIA controls (Mann–Whitney *U *test).

### Impact of glitazones on cartilage

**Figure 5:**
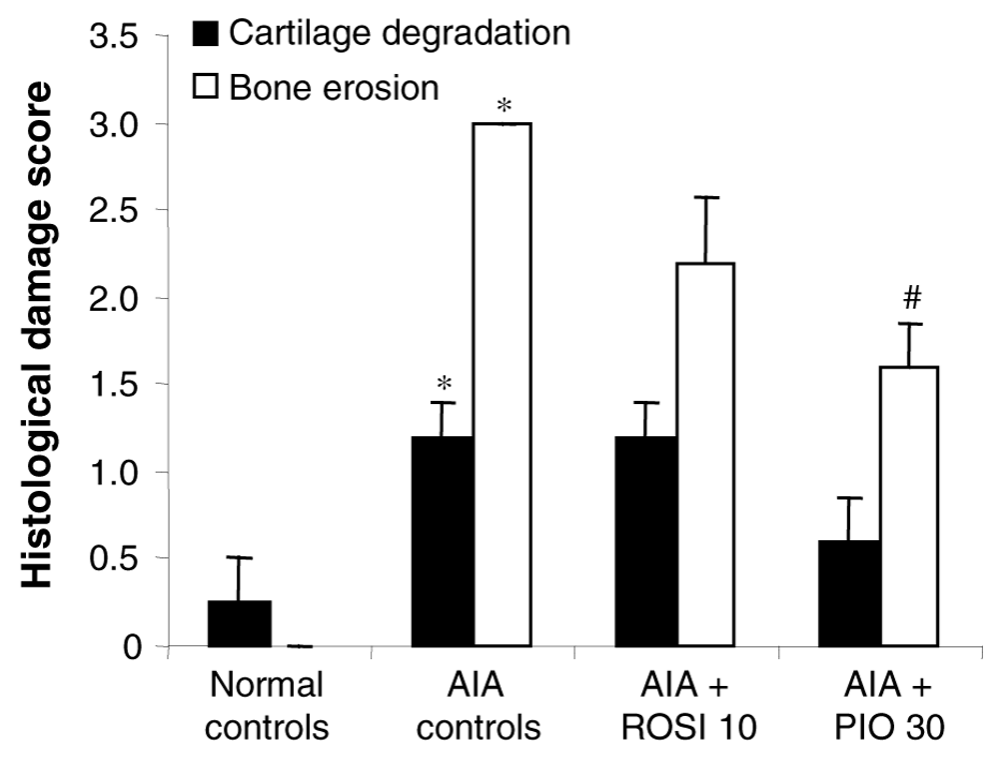
Effect of rosiglitazone and pioglitazone treatment on histological grading of ankle lesions during adjuvant-induced arthritis. Animals were treated daily for 21 days with rosiglitazone (ROSI) 10 mg/kg (*n *= 8) or pioglitazone (PIO) 30 mg/kg (*n *= 8) by oral administration. Arthritic (adjuvant-induced arthritis (AIA)) (*n *= 7) and normal controls (*n *= 7) were given 0.5% carboxymethylcellulose alone. Cartilage degradation and bone erosion were graded as indicated in the Materials and methods section. Data are expressed as means ± SEM for five representative animals per group. *, *P *< 0.05 compared with normal controls; ^#^, *P *< 0.05 compared with AIA controls (Mann–Whitney *U *test).

As shown in Figure 4b, the content of glycosaminoglycans, an indicator of turnover of proteoglycans, was decreased by 17% in arthritic controls compared with naive animals. This loss of glycosaminoglycans was not prevented in rats treated with glitazones. As shown in Figure 4c, radiolabelled sulphate incorporation, an indicator of new proteoglycan synthesis, was markedly decreased in the central and peripheral areas of the patella in arthritic controls. Once again, arthritis-induced inhibition of proteoglycan synthesis was not significantly reduced in rats treated with 10 mg/kg/day of rosiglitazone or 30 mg/kg/day of pioglitazone. RT-PCR analysis showed that aggrecan expression was also downregulated in tibial plateaux of arthritic controls, but a return to normal levels was not observed in rats treated with glitazones (Figure 4d). Finally, histological examination of ankle joints from arthritic controls revealed limited cartilage degradation, characterized mainly by a loss of proteoglycan staining. In contrast, AIA controls exhibited severe bone changes, with erosions at the synovium margin and bone loss (Figure 5). Cartilage lesions were not decreased in rats receiving glitazones, although a trend was observed for pioglitazone at 30 mg/kg/day. In contrast, both rosiglitazone and pioglitazone prevented bone erosion (Figure 5).

### Effect of glitazones on bone metabolism

#### Bone mineral content and fat mass percentage

**Figure 6:**
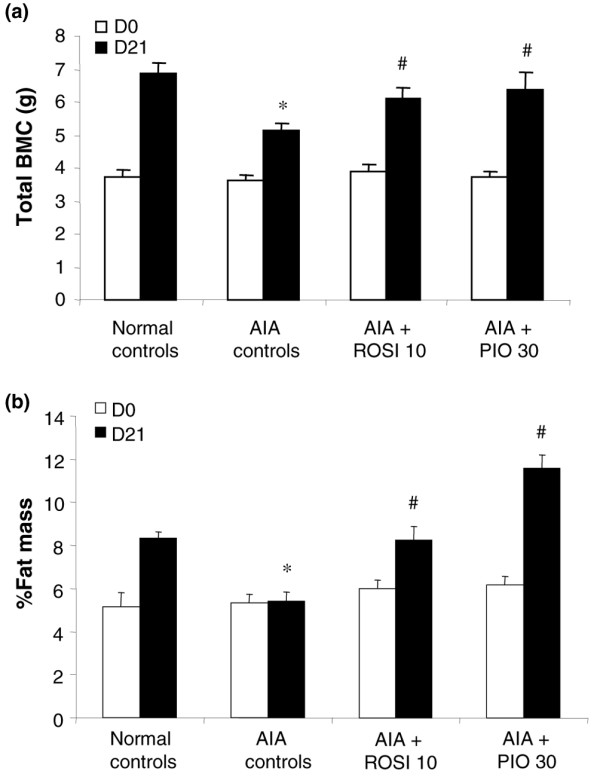
Effect of rosiglitazone and pioglitazone treatment on dual-energy X-ray absorptiometry changes during adjuvant-induced arthritis. Animals were treated daily for 21 days with rosiglitazone (ROSI) 10 mg/kg (*n *= 8) or pioglitazone (PIO) 30 mg/kg (*n *= 8) by oral administration. Arthritic (adjuvant-induced arthritis (AIA)) (*n *= 7) and normal controls (*n *= 7) were given 0.5% carboxymethylcellulose alone. Dual-energy X-ray absorptiometry analysis was performed *in vivo *the day before arthritis induction (day 0) and before necropsy (day 21). **(a) **Changes in whole-body bone mineral content (BMC); **(b) **changes in fat mass percentage. Data are expressed as means ± SEM. *, *P *< 0.05 compared with normal controls; ^#^, *P *< 0.05 compared with AIA controls (ANOVA and Fisher's PLSD post-hoc test). D*n*, day *n*.

Changes in BMC and fat mass were evaluated by DEXA on the whole body. As shown in Figure 6, animals had a similar BMC and fat mass ratio before sensitization (day 0). In normal controls, BMC and fat mass ratio increased notably over the study duration, whereas a limited increase in BMC (Figure 6a) and a stagnation of fat mass (Figure 6b) were observed in arthritic AIA controls. The loss of BMC was partly prevented in arthritic animals treated with 10 mg/kg/day of rosiglitazone or 30 mg/kg/day of pioglitazone (Figure 6a). The percentage of fat mass returned towards normal values in arthritic rats receiving 10 mg/kg/day of rosiglitazone but increased over normal controls in rats treated with 30 mg/kg/day of pioglitazone (Figure 6b). These data were consistent with the increase in body weight of pioglitazone-treated rats before arthritis onset (Figure 1) and suggested that the gain in body weight reflected both the reduction of inflammation and the growth of adipose tissue.

#### Biochemical markers of bone turnover

As shown in Table 6, osteocalcin level decreased with time in all groups of animals. The changes over the study duration were not significantly different between the arthritic rats (AIA controls) and the normal rats, and were modified by neither 10 mg/kg/day of rosiglitazone nor 30 mg/kg/day of pioglitazone (Table 6). In contrast, the deoxypyridinoline/creatinine urinary level remained stable in normal controls but increased significantly in AIA controls. Treatment with 10 mg/kg/day of rosiglitazone or 30 mg/kg/day of pioglitazone tended to decrease deoxypyridinoline/creatinine levels, although this did not reach a statistical level of significance (Table 6).

**Table 6:** Effect of PPAR-γ agonists on biochemical markers of bone turnover in rats with adjuvant arthritis

Group	Change in plasma osteocalcin^a ^(ng/ml)	Change in urinary deoxypyridinoline^b ^(nmol/mmol creatinine)
Normal controls	-39 ± 11	-3 ± 6
AIA controls	-50 ± 9	24 ± 7*
AIA + ROSI 10	-37 ± 12	9 ± 4
AIA + PIO 30	-32 ± 16	12 ± 5

PPAR, peroxisome proliferator-activated receptor; AIA, adjuvant-induced arthritis; ROSI 10, rosiglitazone 10 mg/kg/day; PIO 30, pioglitazone 30 mg/kg/day. Data are expressed as means ± SEM (*n *= 5 for deoxypyridinoline; *n *= 6 for osteocalcin).

*, *P *< 0.05 compared with normal controls (ANOVA and Fisher's PLSD test). ^a^Day 21 minus day -1; ^b^day 21 minus day 0.

### Activation of PPAR-γ target genes by thiazolidinediones

**Figure 7:**
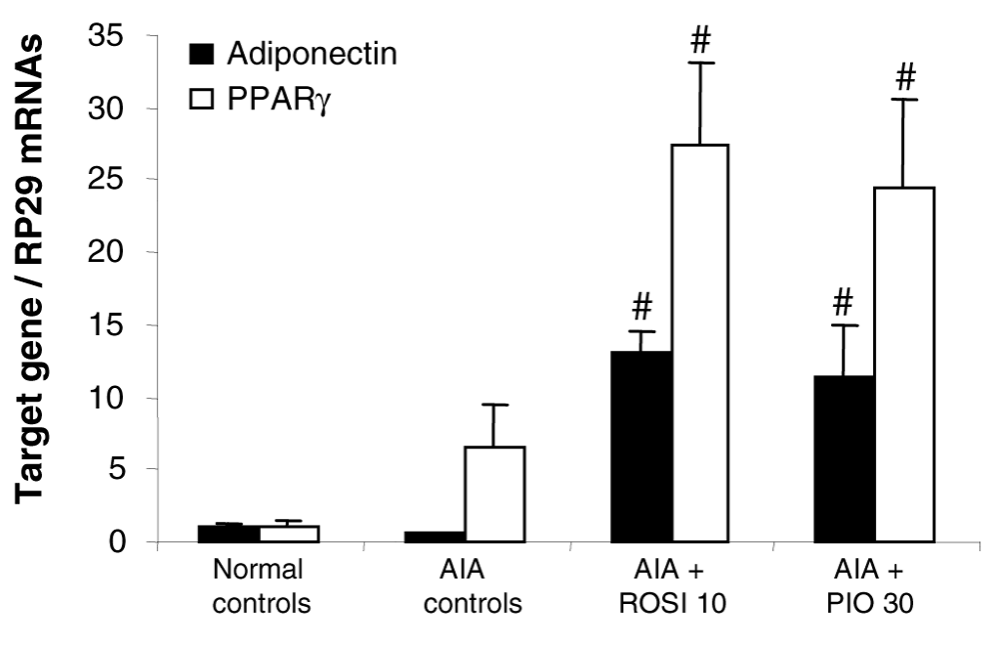
Effect of rosiglitazone and pioglitazone treatment on expression of PPAR target genes during adjuvant-induced arthritis. Animals were treated daily for 21 days with rosiglitazone (ROSI) 10 mg/kg or pioglitazone (PIO) 30 mg/kg by oral administration. Arthritic (adjuvant-induced arthritis (AIA)) and normal controls were given 0.5% carboxymethylcellulose alone. mRNA levels of adiponectin and peroxisome proliferator-activated receptor (PPAR)-γ normalized to RP29 in adipose tissue were assessed by RT-quantitative polymerase chain reaction. Data are expressed as means ± SEM for three representative samples per group (arthritis score close to the mean score of the group). ^#^, *P *< 0.05 compared with AIA controls (ANOVA and Fisher's PLSD post-hoc test).

Figure 7 shows that mRNA levels of PPAR-γ and of adiponectin, a PPAR-γ target gene, were similar in peritoneal adipose tissue of normal or arthritic controls. Expression of both genes was increased in arthritic rats treated with 10 mg/kg/day of rosiglitazone or 30 mg/kg/day of pioglitazone. In the liver, mRNA levels of PPAR-α and of acyl-Coenzyme A oxidase, a PPAR-α target gene, were similar in normal and arthritic controls as well as in rats treated with 10 mg/kg/day of rosiglitazone (data not shown). In rats treated with 30 mg/kg/day of pioglitazone, there was a discrepancy in the induction of these genes, suggesting that the molecule could be less selective for PPAR-γ at its effective anti-arthritic dose (data not shown). These data were consistent with glitazones' being able to induce PPAR-γ-dependent effects in arthritic rats.

## Discussion

In the present study, neither rosiglitazone nor pioglitazone delayed the onset or reduced the incidence of arthritis, suggesting that the immunological spread of the disease was not impaired by the dose regimen used. A previous report showed that PPAR-γ agonists remained anti-arthritic when given after the early sensitization phase of adjuvant arthritis [21], although a decreased immunological response contributed partly to the efficacy of THR0921, a novel PPAR-γ agonist, in mice developing collagen-induced arthritis [39]. The dose-ranging study of TZDs was performed by biotelemetry, which provides a unique opportunity to measure body temperature and locomotive activity continuously in freely moving conscious rodents over the study duration [33]. To some degree, telemetry reproduces the clinical evaluation of RA because locomotive activity contributes to algofunctional indices, whereas fever is not unusual during acute flares [40]. The arthritic animals displayed a biphasic febrile response that was previously shown to reflect inflammation linked to sensitization and immune arthritis, respectively, and to follow the systemic release of pro-inflammatory cytokines [32]. In our experimental model, rosiglitazone and pioglitazone decreased both fever peaks at the highest doses tested but they remained ineffective towards loss of mobility, which is a pain-dependent event [32]. Although TZDs are multistep inhibitors of the inducible arachidonic acid cascade [41], these data do not support a major contribution of prostaglandin E_2_, since cyclo-oxygenase inhibitors were shown to improve the hypomobility of arthritic animals [33].

These anti-inflammatory properties of TZDs may be due to a decreased production of pro-inflammatory cytokines such as TNF-α, IL-1β and IL-6, which have a key role in fever [42] and were shown previously to decrease in the bloodstream of arthritic mice treated with rosiglitazone [23]. In support of this proposal, we demonstrated that the highest doses of TZDs decreased the expression of IL-1β and TNF-α in inflamed synovium, which is a primary source for systemic inflammatory cytokines [3]. The major finding of this dose-ranging study was therefore to indicate that the doses of TZDs required to decrease inflammation were equal to or greater than those sufficient to restore insulin sensitivity, as reported separately for troglitazone [25].

The study of the pathophysiological findings of polyarthritis further demonstrated that rosiglitazone at 10 mg/kg/day and pioglitazone at 30 mg/kg/day decreased most aspects of arthritis severity. These data confirmed that agonists of PPAR-γ are potential therapeutic agents for arthritis [21,23,25,39], although they displayed a variable ability to activate this receptor subtype [39,43]. From that point of view, rosiglitazone was shown to be at least 10-fold more active than pioglitazone in transactivation assays with murine or human PPAR-γ chimeric receptors [43]. In addition, pharmacokinetics studies demonstrated that a single oral dose of 10 mg/kg/day in rats provided a maximal plasma concentration that was twice as high with rosiglitazone [44] as with pioglitazone [45]. In addition, the plasma levels of pioglitazone were shown previously to be lower in male rats than in female rats for a given dose [45]. As a consequence, one might expect that the threefold higher dose of pioglitazone would provide circulating levels in the same range as rosiglitazone in our arthritic male rats. This threefold dose ratio between pioglitazone and rosiglitazone was also consistent with their therapeutic use in patients with type 2 diabetes, although it cannot be considered a reliable indicator of their transactivating properties on PPAR-γ. Of particular note is our result that animals receiving 30 mg/kg/day of pioglitazone had a significantly higher gain in body weight before arthritis onset. To our knowledge, this is the first description of such a side effect in rodents developing arthritis, although it was highly consistent with the weight gain observed in diabetic patients treated with TZDs [46]. Furthermore, the DEXA analysis revealed a higher than normal percentage of fat mass in these animals, which reproduced the increased fatness seen in the clinics, even if a favourable redistribution of fat from visceral to subcutaneous depots was also reported [47]. This drawback was probably attributable to the ability of PPAR-γ to induce adipocyte differentiation [48], because we demonstrated that both TZDs stimulated the expression of adiponectin, a PPAR-γ-sensitive gene [49], in the adipose tissue of arthritic rats. However, the increased fatness was observed only in the group displaying the less severe arthritis. This suggests that a lower caloric cost of the disease could also contribute to the changes in body weight observed in animals treated with 30 mg/kg/day of pioglitazone [50].

The reduction of synovitis in animals treated with 10 mg/kg/day of rosiglitazone or 30 mg/kg/day of pioglitazone extended the general meaning that inflamed synovium was a target for the anti-arthritic potency of TZDs [21-23,25,39]. The decrease in synovial hyperplasia seemed unlikely to have resulted from an increased apoptosis of synovial fibroblasts [25], because rosiglitazone had been shown to decrease apoptotic cells in connected tissues around the joints of arthritic mice [22]. As mentioned previously, we demonstrated that both TZDs reduced the expression of IL-1β and TNF-α in arthritic synovium, a finding consistent with a recent report of their decrease by the PPAR-γ agonist THR0921 in joints of mice with established collagen-induced arthritis [39]. This could be due to the ability of PPAR-γ agonists to inhibit the NF-κB pathway in arthritic tissues [22]. However, differences between arthritis models might explain why we failed to observe any significant decrease in the chemokine monocyte chemoattractant expression in TZDs-treated rats, in contrast with mice treated with THR0921 [39]. We showed further that TZDs decreased the expression of bFGF, which is a powerful mitogen for both synovial fibroblasts and endothelial cells whose neutralization was reported to attenuate arthritis severity [51]. Its reduced expression could therefore contribute to the anti-arthritic potency of these molecules. Finally, we demonstrated that neovascularization and expression of VEGF were reduced by neither pioglitazone nor rosiglitazone, despite a marked decrease in synovitis. Angiogenesis has a pivotal role in RA by increasing the exchange of cells, cytokines and growth factors from the bloodstream to the joint cavity, thereby promoting tissue infiltration and pannus growth [52]. Pro-inflammatory cytokines [53] and bFGF [54] are strong inducers of VEGF release, and their inhibition by TZDs may lower neovascularization in the same way as anti-cytokine therapies in rheumatoid patients [53] or arthritic rats [55]. In contrast, TZDs were reported to stimulate VEGF expression in several cell types [56] and to increase its plasma level in type 2 diabetic patients [57], therefore suggesting that they could counterbalance the impact of their anti-inflammatory effect on the formation of new vessels.

The decrease in ^35^S-sulphate incorporation into proteoglycans [58] and secondary loss of proteoglycan content in cartilaginous tissue [59] are hallmarks of the chronic phase of adjuvant arthritis. In addition, biochemical changes in patellar cartilage were shown to be representative of the entire arthritic joint [60] and to be very sensitive to the inhibitory effect of pro-inflammatory cytokines [61]. Thus, our data were highly homogeneous because the decreased proteoglycan synthesis and aggrecan expression in cartilage resulted in a significant loss of proteoglycans and decreased cartilage staining in arthritic controls. However, the anti-inflammatory effect of TZDs failed to provide significant protection to cartilage in both the knee and ankle joints. Our results differ from previous reports of decreased histological lesions in arthritic joints of rodents treated with glitazones [23,25], although this difference could be supported, at least in part, by the scoring system used. Indeed, cartilage and bone changes were always assessed concomitantly [23,25], whereas we checked separately for cartilage [35] and bone [36] changes in our arthritic rats. Had we considered both changes together, we would have demonstrated a possibly protective effect of TZDs in arthritic joints but it would have been supported by an improvement in bone lesions rather than in cartilage lesions. In addition, we demonstrated recently that selective agonists of the three PPAR isotypes decreased the anabolic response of chondrocytes to TGF-β [62]. Because rat chondrocytes embedded in alginate beads are close to cartilage samples [63], one cannot exclude the possibility that the lack of restoration of proteoglycan metabolism by TZDs could be also supported by a diminished response of arthritic cartilage to TGF-β.

A major finding of the present work was that rosiglitazone and pioglitazone prevented arthritis-induced bone loss. PPAR-γ agonists were shown previously to decrease bone erosion [23,24] and to improve the radiographic score [24] in arthritic rodents. The contribution of synovitis to bone loss, ranging from focal erosions to periarticular or generalized osteopenia, has become a very active topic in RA [24]. Further confirming histological findings, the DEXA analysis showed that the protective effect of TZDs was detectable on the whole body and in selected regions of interest (data not shown), suggesting that they were active on cortical and trabecular bone. Bone loss is a classical feature of adjuvant arthritis, in which an increase in the number of osteoclasts and in the formation of osteoclast precursors from the monocyte/macrophage compartment in the synovium was reported within few days after disease onset [64]. Focal bone erosions and generalized bone loss are thought to be secondary to an overload of pro-inflammatory cytokines [6], whose osteoclastogenic effect has recently been ascribed to RANKL [10]. Thus, administration of osteoprotegerin, which prevents the binding of RANKL to RANK, decreased arthritis-induced bone loss [65], suggesting that TZDs may prevent bone loss, at least in part, by decreasing the production of inflammatory cytokines in inflamed synovium. In additon, PPAR-γ agonists were shown to decrease the phosphorylation of the inhibitor IκB in arthritic joints [22], and inhibition of the NF-κB pathway prevented inflammatory bone destruction *in vivo *by blocking osteoclastogenesis [66]. Moreover, PPAR-γ agonists were shown to inhibit RANKL-dependent differentiation of osteoclasts in bone marrow cells [39,67] or peripheral blood mononuclear cells [21,68]. A decrease in bone resorption was also supported by the decreased urinary excretion of deoxypyridinoline [38] in rats treated with TZDs. The bone-protective potency of TZDs is very provocative because these molecules were reported to cause bone loss in rodents [69,70] or type 2 diabetic patients [71,72] by switching the differentiation of bone precursors towards an adipogenic phenotype [69,70]. Our data therefore suggest that the differentiating effect of PPAR-γ agonists on adipocytes [48] may have a variable impact on bone depending on the inflammatory status of the body.

## Conclusion

The present work confirms that an oral intake of the antidiabetics rosiglitazone or pioglitazone can reduce the severity of arthritis but indicates that the doses required for an anti-arthritic effect exceed those sufficient to restore insulin sensitization. This effect is due, at least in part, to their ability to decrease the expression of pro-inflammatory cytokines in inflamed synovium without affecting neovascularization. Thiazolidinediones prevent inflammatory bone loss but do not improve changes in proteoglycans in arthritic cartilage. However, activation of PPAR-γ in adipose tissue is concomitant with increased adiposity in arthritic animals, suggesting that a strong activation of PPAR-γ may expose arthritic patients to the drawbacks of excessive adipocyte differentiation.

## Abbreviations

ACO = Acyl-CoenzymeA oxidase; AIA = adjuvant-induced arthritis; ANOVA = analysis of variance; bFGF = basic fibroblast growth factor; BMC = bone mineral content; DEXA = dual-energy X-ray absorptiometry; IL = interleukin; MCP-1 = monocyte chemotactic protein-1; NF = nuclear factor; PLSD = protected least-squares difference; PIO = Pioglitazone; PPAR = peroxisome proliferator-activated receptor; RA = rheumatoid arthritis; RANKL = receptor activator of nuclear factor κB ligand; ROSI = rosiglitazone; RT-PCR = polymerase chain reaction with reverse transcription; TNF = tumour necrosis factor; TZD = thiazolidinedione; VEGF = vascular endothelial growth factor.

## Competing interests

The authors declare that they have no competing interests.

## Authors' contributions

MK performed all *in vivo *and molecular studies and drafted the manuscript. DM performed molecular studies and drafted the manuscript. AB and SS performed molecular studies and statistical analysis. MM performed the DEXA analysis. GW performed the DEXA analysis and contributed to the study design. PN supervised the study design and the manuscript. JYJ conceived the study and participated in its design and final presentation. All authors read and approved the final manuscript.
